# Mortality following acute pancreatitis: social deprivation, hospital size and time of admission: record linkage study

**DOI:** 10.1186/1471-230X-14-153

**Published:** 2014-08-28

**Authors:** Stephen E Roberts, Kymberley Thorne, P Adrian Evans, Ashley Akbari, David G Samuel, John G Williams

**Affiliations:** College of Medicine, Swansea University, Singleton Park, Swansea, UK; Department of Emergency Medicine, Morriston Hospital, Swansea, UK; Department of Gastroenterology, Prince Philip Hospital, Llanelli, UK

**Keywords:** Acute pancreatitis, Mortality, Social deprivation, Hospital size, Time of admission

## Abstract

**Background:**

Very little is known about whether mortality following acute pancreatitis may be influenced by the following five factors: social deprivation, week day of admission, recruitment of junior doctors in August each year, European Working Time Directives (EWTDs) for junior doctors’ working hours and hospital size. The aim of this study was to establish how mortality following acute pancreatitis may be influenced by these five factors in a large cohort study.

**Methods:**

Systematic record linkage of inpatient, mortality and primary care data for 10 589 cases of acute pancreatitis in Wales, UK (population 3.0 million), from 1999 to 2010. The main study outcome measure was mortality at 60 days following the date of admission.

**Results:**

Mortality was 6.4% at 60 days. There was no significant variation in mortality according to social deprivation or the week day of admission. There was also no significant variation according to calendar month for acute pancreatitis overall or for gallstone aetiology, but for alcoholic acute pancreatitis, mortality was increased significantly by 93% for admissions during the months of August and September and 102% from August to October when compared with all other calendar months. Mortality was increased significantly for alcoholic aetiology in August 2004, the official month that the first EWTD was implemented, but there were no other increases following the first or second EWTDs. There were also indications of increased mortality in large hospitals when compared with small hospitals, for acute pancreatitis overall and for gallstone aetiology but not for alcoholic acute pancreatitis, although these increases in mortality were of quite marginal significance.

**Conclusions:**

Although we found some evidence of increased mortality for patients admitted with alcoholic acute pancreatitis during August to October, in August 2004, and in large hospitals for acute pancreatitis overall and for gallstone aetiology, the study factors had limited impact on mortality following acute pancreatitis and no significant impact when adjusted for multiple comparisons.

## Background

Acute pancreatitis has become increasingly an important high risk emergency condition. In the UK alone it accounts for approximately 20 000 hospital admissions each year [1, 2]. In recent years, the mortality rate following acute pancreatitis has typically varied between about 4% and 10% [2–9], but increases to about 15% to 30% in cases of severe necrotising pancreatitis [2].

There is large variation in mortality following emergency admissions, including acute pancreatitis, across hospitals in the UK [2, 3, 10]. Although some of this variation is explained by patient case mix, there is major concern that it may be linked to factors such as changes in junior doctor’s working hours, recruitment of newly qualified doctors in August each year, the week day of admission and the hospital size. There have been reductions over time in the working hours of junior doctors through successive European Working Time Directives (EWTDs). The first EWTD officially implemented in the UK on August 1^st^ 2004 reduced working hours from 65 to 56 hours per week, and the second on August 1^st^ 2009 reduced hours further to 48. However, little has been reported as to whether these EWTDs have had any impact on patient outcomes following emergency hospital admissions [11, 12], and nothing has been reported on this for acute pancreatitis. There are concerns about whether the recruitment of newly qualified junior doctors each August affects patient care and outcomes [13], but this has not been reported for acute pancreatitis.

Patient outcomes following acute admissions are often worse when admissions occur at weekends [14–17]. Although the causes are not fully established, reduced staffing levels and access to clinical resources are suspected [14–17]. For acute pancreatitis, the effect of day of admission on mortality has not been reported. Little has also been reported on the relationship between social deprivation and mortality following acute pancreatitis [3]. Studies relating mortality to volume of acute pancreatitis cases have mostly reported lower mortality in hospitals with high volumes of cases, for example, in Sweden [18], the USA [19], Japan [20] and Taiwan [21], but there have been no reports on this from the UK. In a previous study we noted that the hospital admission rate for alcoholic acute pancreatitis was linked positively with consumption of spirits and beer but negatively with wine, and was increased during the Christmas holiday and with high levels of social deprivation [22].

The main objectives of this study were, firstly, to establish the mortality rate following acute pancreatitis in a large population of three million people in Wales, UK. Secondly, to establish whether mortality is influenced by the following five factors; social deprivation, size of hospital, week day of admission, recruitment of newly qualified junior doctors each August and the application of the EWTDs to junior doctors’ working hours.

## Methods

To investigate mortality following hospital admission for acute pancreatitis, we used systematic record linkage of national inpatient, mortality and primary care data across Wales through the Secure Anonymised Information Linkage (SAIL) data [23, 24]. It covers 22 Local Health Authorities and seven National Health Service (NHS) Health Boards for a total population of approximately three million. The inpatient data covers admissions to all NHS hospitals in Wales. In order to identify all deaths that occurred following discharge from hospital along with inpatient deaths, the inpatient data were systematically linked to death certificate data from the Office for National Statistics and the NHS Welsh Administrative Register. To obtain additional information on the aetiology of acute pancreatitis, these inpatient and mortality data were also record linked to SAIL primary care data, obtained from 35% of all general practices across Wales during the entire study period (population 1.0 million). As described and validated elsewhere [23, 24], the record linkage methodology uses a unique, encrypted, anonymised linking field (ALF_E) for each patient which is based, firstly, on the patient NHS number. In cases where the NHS number is absent, other patient identifiers (date of birth, sex, postcode, first name and surname) are used applying a probabilistic matching algorithm MACRAL (Matching Algorithm for Consistent Results in Anonymised Linkage). This methodology has been used as the basis of many studies published in international Medline journals [25–30].

### Ethics statement

Ethical approval and patient consent for the study data (Patient Episode Database for Wales and National Health Service Welsh Administrative Register) were not required. This data is publicly available to other researchers. We were advised by the National Research Ethics Service (NRES) that ethical approval and written patient consent were not required as we were using fully anonymised data, with approval obtained from the Information Governance Review Panel (IGRP) to use this data in the study. The IGRP is represented by NRES, the British Medical Association Ethics Advisor, the Caldicott Guardians and NHS Wales Informatics Service.

### Study inclusion and exclusion criteria

To define the study cohort of patients, we selected admissions where acute pancreatitis was recorded as the principal diagnosis on the discharge record between January 1^st^ 1999 and December 31^st^ 2010. We included each person’s first admission for acute pancreatitis after the start of the study period on January 1st 1999. As acute pancreatitis is often characterised by separate attacks that also require hospitalisation, we included subsequent admissions for acute pancreatitis among the same patients - as ‘new cases’ of acute pancreatitis - if they occurred at least 60 days following discharge after a previous admission. Most of the patients (86%) were hospitalised once only during the 12 year study period. The International Classification of Diseases tenth revision (ICD-10) code used for acute pancreatitis was K85.

### Aetiology of acute pancreatitis

We identified the two main aetiologies of acute pancreatitis (gallstone and alcohol) as described previously [22]. Minor aetiologies were similarly identified using the following ICD-10 codes: hyperlipidaemia (E78), hypercalcaemia (E83.5), malnutrition (E40-E46), abdominal trauma (S30-S39), pancreatic malignancy (C25) and cystic fibrosis (E84).

### Exposure measures

We assessed the impact of the following five factors on mortality: social deprivation, size of hospital, week day of admission, recruitment of newly qualified junior doctors each August and the application of the EWTDs to junior doctors’ working hours.

Social deprivation, was measured using the Welsh Index of Multiple Deprivation (WIMD) 2005 [31], produced by the Welsh Assembly Government. It is compatible with the similar, widely used English Indices of Multiple Deprivation (IMD) [32], and has been used in many publications [22, 25–30]. WIMD is based on postcode residential areas (average population size of 1560) and consists of seven separate (register based) domains or components of deprivation with weighted contributions as follows: ‘income’ (25% contribution), ‘employment’ (25%), ‘education’ (15%), ‘health’ (15%), ‘geographical access to services’ (10%), ‘housing’ (5%) and ‘physical environment’ (5%). The weighted deprivation scores from each component are used to generate an overall social deprivation score, which is then grouped into quintiles of equal population sizes (I = least deprived and V = most deprived quintile).

We investigated the size of the admitting hospital in terms of the total number of beds, defined as ‘small district general hospital (DGH)’ (150–400 beds; six hospitals), ‘medium DGH’ (400–599 beds; nine hospitals), ‘large DGH’ and teaching hospitals (600+ beds; four hospitals) and ‘other small hospitals’ (mainly community or cottage hospitals; <150 beds; 42 hospitals). To assess for any possible effect of the week day of admission, mortality was compared for admissions on weekends (Saturday 00:00 hours to Sunday 23:59 hrs) vs week days vs public holidays.

The recruitment of newly qualified junior doctors, unlike other European countries, occurs almost always in Wales during August. We compared mortality in August with all other months of the year, we then compared mortality in the two months, August and September, and the three months from August to October, with all other calendar months. As other rotation or ‘swap over’ months in the calendar year for junior doctors are in April and December, we also assessed mortality during these months compared with all other months and across each month of the year.

The two EWTDs were not implemented immediately in all hospitals in August 2004 and August 2009, but were mostly introduced more gradually and more variably over time and across hospitals following these two dates. To assess for any possible impact of the EWTDs on mortality following acute pancreatitis, we first assessed monthly trends in mortality over time during the 12 year study period from 1999 to 2010. We then compared mortality during the month, the two months, the three months and the 12 months following the two official implementation dates with mortality during the three years preceding these two dates.

### Patient co-morbidities

When investigating mortality according to the five factors above, we also adjusted for any impact of the following eleven major patient co-morbidities: ischaemic heart disease, other cardiovascular diseases, cerebrovascular disease, other circulatory diseases, malignancies, chronic obstructive pulmonary disease, asthma, diabetes, dementia, liver disease and renal failure. The measurement of these co-morbidities has been described previously [22].

### Methods of analysis

The main study outcome measures were percentage mortality rates at 60 days following admission for acute pancreatitis. Secondary outcome measures were mortality rates at seven and 30 days and hospital admission rates per 100 000 population. 60 day mortality was chosen as the primary outcome measure as 30 day mortality would exclude some deaths that occur during prolonged inpatient stays for severe necrotising cases.

Mortality rates were calculated by dividing the numbers of deaths by the numbers of hospitalised cases and were standardised directly across the month of year using the study population hospitalised with acute pancreatitis. Conventionally, mortality was based on all causes rather than on the underlying cause of death, especially as acute pancreatitis was the certified underlying cause of death in only 61% of cases, which would under represent the actual mortality in the study patients. Causes of death were based on death certificates from the Office for National Statistics. Hospital admission rates were calculated by dividing the number of hospitalised cases for acute pancreatitis by the corresponding Welsh resident populations. They were standardised directly across the month of year, using the direct method and the Welsh population during the study period as the standard, and were expressed per 100 000 population.

The analysis focused on all cases of acute pancreatitis and on the two main aetiologies, gallstone and alcohol separately. Statistical methods included, firstly, logistic regression to establish any impact of the various study exposure measures on mortality following acute pancreatitis. They were adjusted for age group, sex and co-morbidities and were expressed as odds ratios with 95% confidence intervals. Secondly, hierarchical logistic regression was used to investigate mortality when separating the hospital level factor, hospital size, from the other patient level factors. Other methods included logistic regression to investigate any possible trends in mortality rates over the 12 year study period, Spearman’s rank correlations to investigate any association between monthly mortality and hospital admission rates, and the t-test to compare the ages of patients with different aetiologies of acute pancreatitis. Significance was measured at the conventional 5% level (p < 0.05) and also when using a bonferroni correction to adjust for multiple comparisons (p < 0.0009).

## Results

Overall, there were a total of 10 589 cases of acute pancreatitis, involving 8607 different patients. The mean age of the patients at admission was 57.7 years (SD ± 19.2) and a slight majority (4362; 50.7%) were men. 3903 of the cases (36.9%) were of gallstone aetiology and 2327 (22.0%) were of alcoholic aetiology. Other aetiologies or diagnoses included hyperlipidaemia (947; 8.9%), abdominal trauma (161; 1.5%), hypercalcaemia (61; 0.6%), malnutrition (27; 0.3%), pancreatic malignancies (21; 0.2%) and cystic fibrosis (12; 0.1%). In 29.5% of cases (4359) the aetiology was unknown.

Patients with gallstone acute pancreatitis (mean age = 60.6 years; SD ± 18.7) were significantly older (p < 0.001) than those with alcoholic acute pancreatitis (mean age = 43.9 years; SD ± 12.8). Patients with gallstone aetiology were mostly women (60.3%), those with alcoholic acute pancreatitis were predominantly men (77.7%).

### Mortality

Mortality was 3.0% at seven days (based on 315 deaths), 5.6% at 30 days (560 deaths) and 6.4% at 60 days (based on 675 deaths). There was no significant trend in mortality over the 12 year study period (mean annual reduction = 0.2%; p = 0.88). Mortality at 60 days was 5.2% (95% CI = 4.5%-5.9%) for gallstone aetiology and 3.1% (2.4%-3.9%) for alcoholic aetiology (Table 1). For other aetiologies, mortality was 6.9% (5.3%-8.7%) for hyperlipidaemia, 5.0% (2.2%-9.6%) for abdominal trauma, 26.2% (15.8%-39.1%) for hypercalcaemia, 11.1% (2.4%-29.2%) for malnutrition, 19.1% (5.5%-41.9%) for pancreatic malignancies and 0% (0%-26.5%) for cystic fibrosis.

**Table 1:** **Mortality following acute pancreatitis, according to aetiology, age group and sex, 1999 to 2010**

	All cases of acute pancreatitis					Gallstone aetiology					Alcohol aetiology				
Age group	No. of cases*	No. of deaths (30 days)	No. of deaths (60 days)	Mortality rate (%)	(95% CI)	No. of cases	No. of deaths (30 days)	No. of deaths (60 days)	Mortality rate (%)	(95% CI)	No. of cases	No. of deaths (30 days)	No. of deaths (60 days)	Mortality rate (%)	(95% CI)
**Men**															
<35	844	7	8	1.0	(0.4,1.9)	71	1	1	1.4	(0.0,7.6)	435	2	3	0.7	(0.1,2.0)
35-44	958	12	16	1.7	(1.0,2.7)	134	1	1	0.8	(0.0,4.1)	583	10	13	2.2	(1.2,3.8)
45-54	995	22	33	3.3	(2.3,4.6)	224	3	5	2.2	(0.7,5.1)	431	12	16	3.7	(2.1,6.0)
55-64	965	36	44	4.6	(3.3,6.1)	340	10	12	3.5	(1.8,6.1)	250	11	13	5.2	(2.8,8.7)
65-74	898	60	70	7.8	(6.1,9.7)	353	17	20	5.7	(3.5,8.6)	70	1	1	1.4	(0.0,7.7)
75+	925	123	154	16.6	(14.3,19.2)	426	43	58	13.6	(10.5,17.2)	40	5	5	12.5	(4.2,26.8)
All ages	5585	260	325	5.8	(5.2,6.5)	1548	75	97	6.3	(5.1,7.6)	1809	41	51	2.8	(2.1,3.7)
**Women**															
<35	763	1	1	0.1	(0.0,0.7)	389	1	1	0.3	(0.0,1.4)	125	0	0	0	(0.0,2.9)
35-44	563	2	4	0.7	(0.2,1.8)	215	0	0	0.0	(0.0,1.7)	157	1	2	1.3	(0.2,4.5)
45-54	692	12	13	1.9	(1.0,3.2)	313	2	2	0.6	(0.1,2.3)	144	6	6	4.2	(1.5,8.8)
55-64	867	26	38	4.4	(3.1,6.0)	404	5	9	2.2	(1.0,4.2)	55	5	7	12.7	(5.3,24.5)
65-74	802	61	72	9.0	(7.1,11.2)	409	14	23	5.6	(3.6,8.3)	23	2	3	13.0	(2.8,33.6)
75+	1316	198	222	16.9	(14.9,19.0)	625	55	69	11.0	(8.7,13.8)	14	3	3	21.4	(4.7,50.8)
All ages	5003	300	350	7.0	(6.3,7.7)	2355	77	104	4.4	(3.6,5.3)	518	17	21	4.1	(2.5,6.1)
**All patients**	**10 589**	**560**	**675**	**6.4**	**(5.9,6.9)**	**3903**	**152**	**201**	**5.1**	**(4.5,5.9)**	**2327**	**58**	**72**	**3.1**	**(2.4,3.9)**

Notes.

* The sex of one of the 10 589 patients was not recorded.

For 289 fatalities from 2003 onwards for which the causes of death were available, the certified underlying cause of death was acute pancreatitis in 176 cases (61%), gallstones (38; 13%), gastrointestinal malignancies (11; 4%), ischaemic heart disease (10; 3%), other diseases of the pancreas (7; 2%), liver disease (6; 2%) and various other disorders (41; 14%). In the 38 cases where gallstones were certified as the underlying cause of death, the certified immediate cause of death was acute pancreatitis in 30 cases, with other diseases of the pancreas, liver disease, genitourinary disease, post-operative disorders and infections certified in the remaining cases.

### Social deprivation

There were no significant associations overall between social deprivation and mortality for acute pancreatitis overall (Table 2), for gallstone acute pancreatitis or for alcoholic acute pancreatitis (Table 3).

**Table 2:** **Acute pancreatitis: numbers of cases, deaths and mortality rates according to factors, 1999 to 2010**

	All cases of acute pancreatitis				
Factor	No. of cases	No. of deaths		Mortality rate (60 days)	Adjusted 60 day mortality odds ratio † (95% CI)
		30 days	60 days		
**All cases**	10 589	560	675	6.4%	
**Age group:**					
<35	1607	8	9	0.6%	
35-44	1520	14	20	1.3%	
45-54	1688	34	46	2.7%	
55-64	1833	62	82	4.5%	
65-74	1701	121	142	8.3%	
75-84	1540	181	223	14.5%	
85+	702	140	153	21.8%	
**Sex:**					
Men	5584	260	325	5.8%	1.00 Ref
Women	5003	300	350	7.0%	0.91 (0.77, 1.07)
**Social deprivation:**					
I (least deprived)	1638	101	117	7.2%	1.00 Ref
II	1810	99	114	6.3%	0.91 (0.69, 1.20)
III	2118	104	132	6.2%	0.94 (0.72, 1.23)
IV	2256	137	160	7.1%	1.24 (0.96, 1.60)
V (most deprived)	2270	119	155	5.5%	1.06 (0.82, 1.37)
**Size of hospital:**					
Small hospitals	1452	65	82	5.7%	1.00 Ref
Medium hospitals	4537	233	278	6.1%	1.23 (0.95, 1.60)
Large hospitals	3893	228	270	6.9%	1.53 (1.17, 1.98)*
Other hospitals	106	4	9	8.5%	0.90 (0.44. 1.89)
**Week day of admission:**					
Weekday	7803	414	506	6.5%	1.00 Ref
Weekend	2563	136	156	6.1%	0.89 (0.74, 1.08)
Public holiday	226	10	13	5.8%	0.89 (0.50, 1.61)
**Month of admission:**					
Except in August	9623	509	608	6.3%	1.00 Ref
August	966	51	67	6.9%	1.21 (0.92, 1.58)
Except in August – September	8753	456	548	6.3%	1.00
August – September	1836	104	127	6.9%	1.20 (0.98, 1.48)
Except in August – October	7812	396	481	6.2%	1.00
August – October	2777	164	194	7.0%	1.18 (0.99, 1.41)
**First european working time directive (August 2004):**					
Admitted Aug 01 – July 04	2375	149	168	7.1%	1.00 Ref
Admitted Aug 2004	74	4	7	9.5%	1.69 (0.72, 3.93)
Admitted Aug – Sept 04	124	7	10	8.1%	1.10 (0.55, 2.20)
Admitted Aug – Oct 04	188	15	18	9.6%	1.29 (0.75, 2.20)
Admitted Aug 04 – July 05	810	46	58	7.2%	1.00 (0.72, 1.37)
**Second european working time directive (August 2009):**					
Admitted Aug 06 – Jul 09	2938	141	175	6.0%	1.00 Ref
Admitted Aug 2009	93	1	3	3.2%	0.47 (0.14, 1.56)
Admitted Aug – Sept 09	182	2	5	2.7%	0.43 (0.17, 1.08)
Admitted Aug – Oct 09	280	8	11	3.9%	0.62 (0.33, 1.18)
Admitted Aug 09 – July 10	1099	43	57	5.2%	0.86 (0.62, 1.18)

Notes.

† The odds ratio for sex is adjusted for age group. The odds ratios for all other factors are adjusted for age group and sex.

* Denotes significance at the 5% level. P-value = 0.002.

Ref = Reference category.

**Table 3:** **Gallstone and alcoholic acute pancreatitis: numbers of cases, deaths and mortality rates according to factors, 1999 to 2010**

	Gallstone acute pancreatitis					Alcoholic acute pancreatitis				
Factor	No. of cases	No. of deaths		Mortality rate (60 days)	Adjusted 60 day mortality odds ratio † (95% CI)	No. of cases	No. of deaths		Mortality rate (60 days)	Adjusted 60 day mortality odds ratio † (95% CI)
		30 days	60 days				30 days	60 days		
**All cases**	3903	152	201	5.2%		2327	58	72	3.1%	
**Age group:**										
<35	460	2	2	0.4%		560	2	3	0.5%	
35-44	349	1	1	0.3%		674	11	15	2.2%	
45-54	537	5	7	1.3%		575	18	22	3.8%	
55-64	745	15	21	2.8%		305	16	20	6.6%	
65-74	763	31	43	5.6%		93	3	4	4.3%	
75-84	727	55	77	10.6%		45	5	5	11.1%	
85+	324	43	50	15.4%		9	3	3	33.3%	
**Sex:**										
Men	1548	75	97	6.3%	1.00 Ref	1809	41	51	2.8%	1.00 Ref
Women	2355	77	104	4.4%	0.72 (0.53,0.97)*	518	17	21	4.1%	1.46 (0.86, 2.48)
**Social deprivation:**										
I (least deprived)	673	24	34	5.1%	1.00 Ref	245	6	7	2.9%	1.00 Ref
II	746	24	29	3.9%	0.76 (0.45, 1.27)	273	9	11	4.0%	1.74 (0.65, 4.69)
III	824	31	43	5.2%	1.08 (0.67, 1.73)	359	13	13	3.6%	1.61 (0.61, 4.40)
IV	768	37	49	6.4%	1.43 (0.90, 2.27)	571	19	23	4.0%	1.80 (0.78, 4.64)
V (most deprived)	894	36	46	5.1%	1.34 (0.84, 2.14)	879	11	18	2.0%	0.95 (0.38, 2.57)
**Size of hospital:**										
Small hospitals	612	20	26	4.3%	1.00 Ref	240	3	5	2.1%	1.00 Ref
Medium hospitals	1588	59	75	4.7%	1.27 (0.80, 2.02)	942	27	32	3.4%	1.67 (0.63, 4.18)
Large hospitals	1419	65	86	6.1%	1.69 (1.07, 2.67)*	1029	26	31	3.0%	1.39 (0.53, 3.66)
Other hospitals	39	1	3	7.7%	1.13 (0.32, 3.97)	14	0	1	7.1%	1.74 (0.17, 18.2)
**Week day of admission:**										
Weekday	2860	116	153	5.3%	1.00 Ref	1226	41	52	4.2%	1.00 Ref
Weekend	963	34	45	4.7%	0.83 (0.59, 1.18)	535	15	16	3.0%	0.89 (0.50, 1.60)
Public holiday	82	2	3	3.7%	0.73 (0.22, 2.40)	66	2	4	6.1%	1.84 (0.62, 5.47)
**Month of admission:**										
Except in August	3555	141	183	5.1%	1.00 Ref	2103	49	60	2.9%	1.00 Ref
August	348	11	18	5.2%	1.15 (0.69, 1.92)	224	9	12	5.4%	1.87 (0.96, 3.63)
Except in August –Sept	3258	131	168	5.2%	1.00 Ref	1911	40	51	2.7%	1.00 Ref
August – Sept	645	21	33	5.1%	1.07 ( 0.72, 1.59)	416	18	21	5.0%	1.93 (1.13,3.03)*
Except in August-October	2905	121	157	5.4%	1.00 Ref	1707	31	41	2.4%	1.00 Ref
August – October	998	31	44	4.4%	0.84 (0.59, 1.19)	620	27	31	5.0%	2.02 (1.23,3.29)*
**First European Working Time directive (Aug 2004)**										
Admitted Aug 01 – July 04	797	34	42	5.3%	1.00 Ref	479	8	13	2.7%	1.00 Ref
Admitted Aug 2004	27	1	3	11.1%	3.68 (0.93, 14.6)	27	2	2	7.4%	7.49 (1.25,44.8)* *
Admitted Aug – Sept 04	46	2	4	8.7%	1.76 (0.56, 5.51)	38	2	2	5.3%	3.91 (0.70, 22.1)
Admitted Aug – Oct 04	66	4	6	9.1%	1.90 (0.73, 4.96)	51	2	2	3.9%	2.80 (0.52, 15.0)
Admitted Aug 04 – July 05	304	10	16	5.3%	1.11 (0.60, 2.06)	182	6	6	3.3%	1.77 (0.61, 5.19)
**Second European Working Time directive (Aug 2009)**										
Admitted Aug 06 – Jul 09	1154	49	65	5.6%	1.00 Ref	711	14	18	2.5%	1.00 Ref
Admitted Aug 2009	27	0	0	0%		23	0	0	0%	
Admitted Aug – Sept 09	66	0	1	1.5%	0.26 (0.04, 1.95)	40	0	0	0%	
Admitted Aug – Oct 09	105	1	2	1.9%	0.33 (0.08, 1.41)	67	1	1	1.5%	0.46 (0.06, 3.70)
Admitted Aug 09 – July 10	426	14	20	4.7%	0.81 (0.48, 1.37)	281	5	7	1.8%	0.92 (0.37, 2.30)

Notes.

† The odds ratio for sex is adjusted for age group. The odds ratios for all other factors are adjusted for age group and sex.

* Denotes significance at the 5% level. Significant p values for gallstone acute pancreatitis are as follows: women = 0.032; large hospitals = 0.025.

** Denotes significance at the 1% level.

Significant p values for alcoholic acute pancreatitis are as follows: August - Sept = 0.017; August - October = 0.004; Admitted Aug 2004 = 0.027.

Ref = Reference category.

### Size of hospital

In the four large hospitals, compared with small hospitals, mortality was increased significantly by 53% for all cases of acute pancreatitis (Table 2), significantly by 69% for gallstone aetiology, but there was no significant increase (39%) for alcoholic aetiology (Table 3). Mortality in medium size was broadly similar to that in small hospitals. The median length of stay was the same in small, medium and large hospitals (6 days), but was significantly higher (p < 0.001) in other small (mainly community and cottage) hospitals (13 days). There were a total of 267 transfers out of the 10,589 cases (2.5%), including Emergency Department transfers as well as inpatient transfers. 95 patients (0.9%) were transferred to the four large centres, with a mortality rate of 20% (19 deaths).

### Week day of admission

There were no significant differences in mortality according to the week day of admission (Tables 2 and 3), although there was a (non-significant) increase of 84% for alcoholic pancreatitis admissions on public holidays, compared with normal week days (Table 3).

### Month of admission and recruitment of junior doctors each August

**Figure 1:**
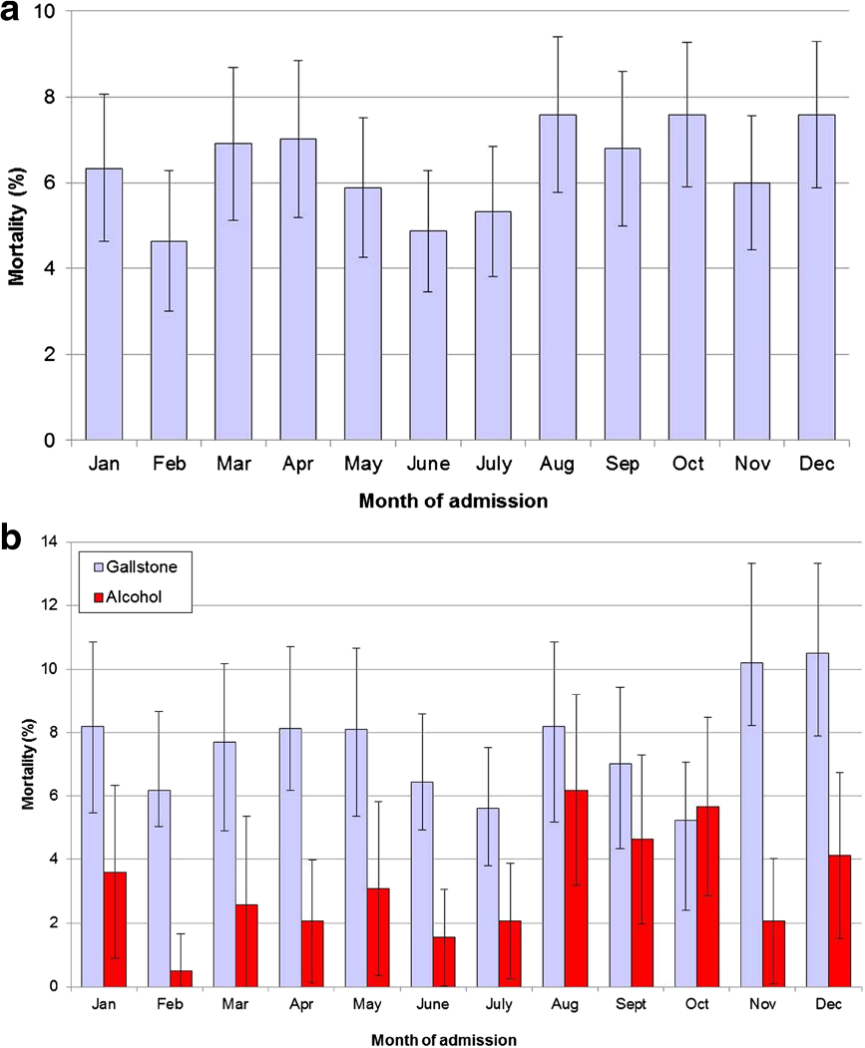
**Mortality following acute pancreatitis, according to the month of admission, 1999 to 2010. a).** All acute pancreatitis. **b).** Gallstone and alcoholic acute pancreatitis. Notes. Mortality rates are standardised directly for age group and sex. Vertical bars represent 95% confidence intervals.

For acute pancreatitis overall, there were indications that mortality was increased for admissions in the months of August (by 21%), August and September (by 20%) and August to October (by 18%) compared with admissions during all other months of the year (Table 2; Figure 1a), although these increases were marginally non-significant. For gallstone aetiology there were also no significant increases in mortality during these months (Table 3, Figure 1b). For alcoholic acute pancreatitis, mortality was increased significantly (p < 0.05) by 93% for admissions during August and September, significantly (p < 0.05) by 102% for admissions in August to October and (non-significantly) by 87% for admissions in August (Table 3; Figure 1b). Regarding other rotation months for junior doctors in April and December each year, overall mortality was relatively high during these two months (7.1% and 7.7% respectively; Figure 1a). Although it was not significantly increased during these months when compared with all other 11 months of the year, mortality was significantly increased (p < 0.05) when April and December were combined and compared with the other 10 months of the year.

### European working time directives

**Figure 2:**
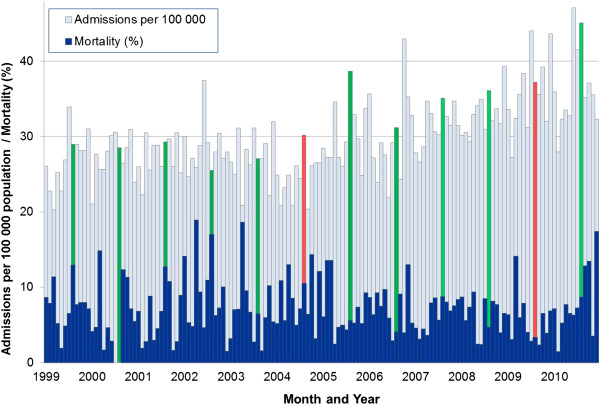
**Mortality and admissions for acute pancreatitis, according to the month and year, 1999 to 2010.** Notes. Red columns denote the two European Working Time Directives in August 2004 and August 2009. Green columns denote the recruitment of newly qualified junior doctors in August each year. Vertical bars represent 95% confidence intervals.

Following the first EWTD in August 2004, there were indications that mortality was increased during subsequent months (Figure 2; Tables 2 and 3) although this was significant (p < 0.05) only for alcoholic acute pancreatitis (7.5 fold increased risk of mortality; during the month of implementation (August 2004) compared with mortality in the preceding three years (Table 3). For the second EWTD, mortality was low (3.2%) during the official month of implementation, August 2009). Although it then appeared to increase in the months subsequent to August 2009 (Figure 2), there was no significantly increased risk at months one, two, three or twelve following the second EWTD (Tables 2 and 3). Over the 12 year study period there was no significant correlation between monthly mortality and the monthly admission rate (Figure 2) for acute pancreatitis (Spearman’s rank correlation = −0.085; p = 0.31). When using hierarchical logistic regression to separate hospital size from the patient level factors, this made little difference to the study findings. For gallstone aetiology, the reduced mortality odds ratio for women changed from 0.72 (0.53-0.97) to 0.71 (0.53-0.95) and for alcohol aetiology the odds ratios for admissions in August to September and August to October changed from 1.93 (1.13-3.03) and 2.02 (1.23-3.29) to 1.95 (1.14-3.34) and 2.06 (1.26-3.37). When using a bonferroni correction to adjust for the multiple comparisons in the study (p < 0.0009), none of the study factors were significant.

### Adjustment for patent co-morbidities

The increased mortality reported above was similar after adjusting for patient co-morbidities. For all cases of acute pancreatitis (Table 2), after adjusting for patient co-morbidities, the increased mortality for large hospitals compared with small hospitals reduced slightly from 1.53 (95% CI = 1.17-1.98) to 1.42 (1.08-1.87). For gallstone acute pancreatitis (Table 3), after adjustment, the increased mortality for large hospitals compared with small hospitals was almost unchanged (1.69 to 1.66) and the reduced mortality for women changed from 0.72 (0.53-0.97) to 0.99 (0.83-1.18). For alcoholic acute pancreatitis (Table 3), the increased mortality for admissions during August, August and September and August to October increased slightly from 1.87 (95% CI = 0.96-3.63), 1.93 (1.13-3.03) and 2.02 (1.23-3.29) respectively to 2.20 (1.12- 3.41), 2.07 (1.19-3.58) and 2.14 (1.30-3.55) and the increased mortality for admissions during August 2004 (first EWTD) reduced from 7.49 (1.25-44.8) to 6.23 (0.88-43.9). The significance of all other factors in Tables 2 and 3 was unaffected by adjustment for patient co-morbidities.

### First and recurrent attacks of acute pancreatitis

When confining the study to first attacks of acute pancreatitis only, rather than also including subsequent attacks, the mortality rates at 60 days were respectively 7.3% (95% CI = 6.7, 7.8) for all cases of acute pancreatitis, 5.3% (4.6, 6.1) for gallstone aetiology and 3.9% (3.0, 5.0) for alcohol aetiology. When considering first attacks only, the increased mortality at 60 days for large hospitals compared with small hospitals (Table 2) increased slightly from 1.53 (1.17-1.98) to 1.60 (1.20, 2.10). The significance of all other factors was unaffected, except social deprivation quintile IV (compared with I) which increased slightly from 1.24 (0.96-1.60) to 1.32 (1.01-1.73).

For gallstone aetiology, when confining the study to first attacks, the significantly reduced mortality for females compared with males was unaffected (0.72 to 0.71) and the significantly increased mortality for large vs small hospitals increased slightly from 1.69 (1.07-2.67) to 1.81 (1.11-2.94). For alcohol aetiology, the reduced mortality for admissions during August, August and September, and August to October changed from 1.87 (0.96-3.63), 1.93 (1.13-3.03) and 2.02 (1.23-3.29) respectively to 2.10 (1.00-4.42), 2.39 (1.31-4.34) and 1.99 (1.34-3.50). The increased mortality for social deprivation quintile IV compared with I increased from 1.80 (0.78-4.64) to 3.32 (1.02-10.79) and the increased mortality for admissions during August 2004 (first EWTD) fell from 7.49 (1.25-44.8) to 5.29 (0.88-31.83). The significance of all other factors was unaffected.

## Discussion

Major strengths of the study are that it provides new evidence on how five factors – social deprivation, size of hospital, day and month of admission, and EWTDs – are related to patient mortality following acute pancreatitis. Secondly, it is a large study, covering more than 10 000 cases of acute pancreatitis. It is based on systematic, validated record linkage of inpatient, death certificate and primary care data to identify all admissions and all deaths that occur following discharge from hospital as well as those that occur in hospital. Previous studies have shown that the principal inpatient diagnoses used in this study for other gastrointestinal conditions (ulcerative colitis and Crohn’s disease), the ascertainment of mortality and the record linkage methodology are respectively >90%, >98% and >99.8% accurate [24, 33].

Study limitations are, firstly, that the study was restricted to NHS hospitals, although the private hospital sector in Wales is very small and receives few admissions for emergency conditions such as acute pancreatitis. Secondly, as with other large-scale studies of acute pancreatitis that have used administrative health data [3–8, 19, 20, 22], the study lacks detailed information about disease history, alcohol consumption, tobacco use, body mass index, pathology, case severity and treatment. Thirdly, through using administrative health data, we included people who were specifically diagnosed with acute pancreatitis at discharge but excluded a small number of cases (<2.6%) of acute pancreatitis that were recorded less specifically as ‘disease of pancreas, unspecified’. As in other studies of acute pancreatitis that have used administrative data [3–8, 19, 20, 22], the ascertainment of aetiologies from recorded patient diagnoses was incomplete in a minority of cases. Also, as with most of these other studies,[3–6, 19, 20, 22] our first identified cases of acute pancreatitis occurred following the start of the study period and we acknowledge that some may have occurred late in the natural history of disease. However, since 86% of people were hospitalised with one attack only during the twelve year study period, this would affect a minority of patients. As per convention [3–8, 19–22, 26–30], we included deaths from all causes when investigating mortality. Our mortality rate of 6.4% at 60 days is comparable with that in other European studies; for example: 6.7% in England from 1998 to 2005 [3], 6.2% in Sweden from 1998 to 2003 [8], and 7.5% in North Jutland, Denmark from 1981 to 2000 [4].

### Social deprivation

We found no significant association between mortality and social deprivation. The only previous study that has reported on social deprivation and mortality following acute pancreatitis - in England from 1998 to 2005 - found a consistent mortality gradient across quintiles I to V and a significant but moderate 19% increased risk for quintile V compared with I [3]. It is possible that this may reflect smaller differences across social deprivation quintiles in Wales.

### Size of hospital

In the four large hospitals, compared with small hospitals, we found some evidence of increased mortality of 53% for acute pancreatitis overall and 69% for gallstone aetiology, but comparable mortality between small and medium size hospitals. This contrasts with studies internationally that have often reported reduced mortality in hospitals with high volumes of cases of acute pancreatitis [18–21]. Better patient outcomes have also been reported for hospitals and surgeons with high volumes of cases [34, 35]. We found that the evidence of increased mortality in the large hospitals could not be attributed to differences in case mix and case severity measures such as co-morbidities and length of stay, nor to possible transfers of severe cases to large hospitals. Unlike other European countries, pre-admission triage based on case severity of acute pancreatitis is seldom implemented in our population. Our findings could reflect differences in configuration of health services compared with other countries and may indicate that specialisation towards surgery and treatment of more complex disorders in some large hospitals could lead to lower priorities for more common emergency disorders among older people. It is however somewhat a surprising finding where the cause is gallstone, as specialist endoscopic investigation and treatment is indicated to identify and extract the stone at an early stage. However, our finding should be regarded only as a possible indicator, since it was of quite marginal significance and since there were only four ‘large’ hospitals in our study population.

### Week day of admission

We found no significant increase in mortality for admissions on weekends or public holidays, when compared with normal week days, although there were indications of an 84% increase in mortality for admissions on public holidays for alcoholic acute pancreatitis. Internationally, many but not all studies have reported increased mortality for admissions on weekends for high risk acute disorders such as myocardial infarction [14], stroke [15, 16], aortic aneurysm rupture [36], upper gastrointestinal bleeding [27, 37] and for all emergency conditions [17], although most increased risks were quite moderate, ranging from 5% to 20%. This indicates that these modest increased risks for emergency admissions on weekends may often be attributable largely to major high risk conditions. The suggestion of higher mortality on public holidays than on weekends is consistent with previous UK studies that have identified increased mortality of >40% on public holidays compared with <15% increased mortality weekends for upper gastrointestinal bleeding [27], and for all emergency admissions [38].

### Month of admission and recruitment of junior doctors each August

We found evidence of increased mortality for alcoholic acute pancreatitis during the months from August to October, but no significant increase for acute pancreatitis overall or for gallstone acute pancreatitis. It is possible that an increase in mortality during popular holiday months such as August and September may be linked to a lack of senior consultant cover and low senior to junior doctor ratios, as well as the recruitment of newly qualified junior doctors in August. It is also possible that the increased mortality for patients with alcoholic aetiology from August to October could be linked to higher alcohol consumption and case severity, particularly during the main summer holiday month of August when mortality was highest for alcohol aetiology. Alcohol aetiology pancreatitis may receive lower priority care during these months, as reported for other alcoholic conditions such as alcoholic liver cirrhosis [39].

However, the limited overall association between mortality and admissions during August could be affected to some extent by other ‘swap over’ points in the calendar year, during December and April. We found relatively high mortality during these two months (7.1% and 7.7% respectively). A previous UK study that investigated mortality according to the recruitment of newly qualified doctors each August, reported a significant 6% increase for all emergency admissions during the first Wednesday in August, compared with the previous Wednesday [13]. In other countries, similar March or July effects have been reported to have little or moderate impact on patient mortality [40–42].

### European working time directives

We found little evidence overall that the two EWTDs have had a significant impact on patient mortality following acute pancreatitis up to the end of 2010, although mortality was increased during August 2004 for alcoholic acute pancreatitis. Two previous studies have reported no major impact of EWTDs on patient outcomes following acute admissions [11, 12]. However, several studies have documented adverse effects of EWTDs on continuity of patient care and training time [43, 44], particularly for surgical specialties, which manage most acute pancreatitis patients. EWTD reductions over time in working hours should have a positive impact on reducing fatigue among junior doctors and patient care. Nonetheless, concerns about the implementation of EWTDs and staffing rotas are reduced numbers of doctors on shifts, fragmentations of teams caring for patients, increased handovers, and greater numbers of juniors working alone out of hours, with potentially adverse impacts on patient safety and outcomes. Further studies are required to investigate their impact on patient outcomes. Although the study findings do not show strong associations between mortality following acute pancreatitis and social deprivation, hospital size and timing of admission, more ‘negative findings’ are important to balance possible publication biases towards ‘positive findings’ in meta analyses of factors that affect patient mortality.

## Conclusions

To summarise, for alcoholic acute pancreatitis, we found significantly increased mortality during the months from August to October when compared with other calendar months of the year, and also during the month of the first EWTD for junior doctors’ working hours (August 2004). There were indications of increased mortality during junior doctors’ rotation months (April and December) and also in large hospitals, compared with small hospitals, for acute pancreatitis overall and for gallstone aetiology but there was no increase for alcoholic acute pancreatitis. However, these increases in mortality were of quite marginal significance. The study found no other significant associations between mortality following acute pancreatitis and the two EWTDs nor with social deprivation or the week day of admission. Overall, the factors studied had limited impact on mortality following acute pancreatitis and no significant impact when adjusting for multiple comparisons.

## Abbreviations

EWTD: European working time directive
NHS: National health service
DGH: District general hospital.
